# Personalized Dietary Self-Management and Its Influence on Disease Progression in Chronic Hepatitis B

**DOI:** 10.1155/jnme/5585004

**Published:** 2025-07-17

**Authors:** Yuan-Yuan Wang, Yu-Qian Yao, Yue Sun, Xiang-Yun Qian

**Affiliations:** ^1^Nantong Third People's Hospital, Nantong, China; ^2^Nantong University College of Medicine, Nantong, China

**Keywords:** chronic hepatitis B, dietary self-management, personalized care, vegetable oil

## Abstract

**Background:** Clinical treatment of chronic hepatitis B (CHB) patients nowadays is still focusing on the clearance ratio of HBsAg. However, the quality of the CHB patients' lives and the recovery of their liver organs also need to be considered in the clinic, especially in the nursing field. Here, we evaluated a newly personalized dietary self-management, which emphasized vegetable oil rather than the oneness requirement of a low-oil diet for the patients, accommodating the thinner patient group for their sufficient energy intake.

**Method:** An observational study was conducted with 90 individual CHB patients through the double-arm randomized study method. The newly personalized dietary self-management education was performed among the participants in the refined group, and their physiological detection results after 6 months from enrollment would be compared with those of the control group participants who received traditional dietary self-management education.

**Result:** Compared to the control group with traditional dietary self-management education, we found that the results in the refined group presented a faster reduction speed in ALT, AST, and TBIL.

**Conclusion:** The results of this study showed the benefit of the vegetable oil for CHB patients when it appropriately served as the way of energy intake, during the patients' treatment period. A larger scale of this personalized dietary self-management education should be permitted for further assessment.

## 1. Introduction

Chronic hepatitis B (CHB) remains a significant global health problem in clinics. Without effective treatments or clinical nursing, CHB in 15%–40% of the CHB virus (HBV)–infected persons will be aggravated to cirrhosis and even worse to liver failure and liver cancer [1–3]. The majority of the CHB patients have to experience a long period of medical therapy. While the increasing studies addressed that the clinical care of those patients during this long period presented equal importance as the drug treatment in the prevention of CHB disease progression from deterioration [4, 5].

An unhealthy diet would contribute to the metabolic burden of the liver and might accelerate the progression of liver disease [6, 7]. The unhealthy diet includes the high-fat diet that has been commonly researched for its relationship with nonalcoholic fatty liver disease, which has rarely been investigated in the progression of CHB-induced liver cirrhosis, however. However, in the animal model, the high-fat diet exhibited the promotion of aging and aggravation of metabolic burden in a recent study, which disclosed the relationships between lipid metabolism and liver recovery [8]. Furthermore, a previous research demonstrated that a high-fat diet was able to induce HBV chronic infection concomitant with nonalcoholic steatohepatitis (NASH) in the mouse model [9]. The controversial point is that the food with oil has been regarded as high-fat absorption in the traditional sense, indicating the deterioration of liver condition in CHB patients. However, the oil from vegetables contains more unsaturated fatty acids, which have been proven to be related to anti-inflammation and are useful in protecting the liver from alcohol injury [10, 11]. On the other hand, appropriate oil/fat and carbohydrate were the vital energy sources for the asthenic patients to recharge their strength [12–15]. Based on this, we became interested in the new personalized self-management strategy of the vegetable oil diet for exploring whether the lipid intake from vegetable oil had a better effect than the intercept of any oil intake to those CHB patients on disease remission with clinical care.

Hence, we designed a novel personalized care project of dietary self-management that restricts the oil intake for obesity CHB patients but recommends moderate vegetable oil intake for the normal and asthenic CHB patients. Then, we compared the half-year effects of CHB treatment results between the personalized vegetable oil dietary group and the traditional low-oil dietary group. The assessment data revealed that the personalized vegetable oil dietary self-management had contributed to the reduction of alanine aminotransferase (ALT) and aspartate aminotransferase (AST) values but did not present the effects on the clearance rate of HBsAg at present. These results indicated that an appropriate vegetable oil diet should be beneficial to the CHB patients in the clinic with the promotion of liver recovery during the treatment period.

## 2. Methods and Analysis

### 2.1. Study Design

**Figure 1:**
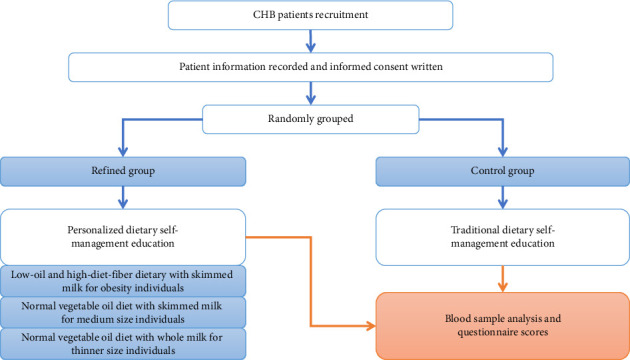
Strategy of the trial process and the study design of the dietary self-management.

This study is a double-arm randomized observational study with a refined dietary education plan on the small cohort (45 individuals), comparing the parallel cohort (45 individuals) with traditional dietary education, to assess whether the newly personalized dietary self-management could improve the life quality of the CHB patients. The detailed process is shown in Figure 1. Each participant recruited in this study was arranged in the two groups randomly and unbiasedly. Members of the refined group had received new personalized dietary self-management education, while those of the control group were educated by the traditional but also current dietary self-management rules, which were based on the standard of behavioral modification [16] and the schedule recommended in a previous study [17]. Within the refined group's personalized dietary self-management education, overweight participants continued to receive the standard intervention: a prescribed low-fat, high-dietary fiber diet. For normal-weight and underweight participants, moderate fat intake was recommended, with fats sourced exclusively from vegetable oils (such as soybean, olive, flaxseed, and palm oil). Additionally, underweight participants were advised to consume whole milk to ensure adequate dietary fat intake for metabolic balance. The detailed schedule of refined items is demonstrated in Table 1. All the participants received clinical treatment for CHB normally. After 2 months of dietary education and another 4 months of telephone reminders, all the participants were asked to complete questionnaires. The data of their blood samples were obtained at the last time of the routine pathological analysis in clinical treatments, including ALT, AST, TBIL, and HBsAg assay (see Figure 1).

### 2.2. Randomization

This study employed a simple randomization method to allocate 90 eligible patients to either the control group (*n* = 45) or the refined group (*n* = 45). The specific allocation procedure was as follows: All patients were assigned sequential numbers (1–120) based on their outpatient visit order. Using the RANDBETWEEN function in Excel software, a random number was generated for each patient, with “1” designating the refined group and “2” designating the control group. A statistician independent of the research team generated the random allocation sequence in advance. The results were sealed within opaque envelopes labeled with the corresponding patient numbers. These envelopes were distributed sequentially according to patient number. Upon enrollment, the researcher opened the assigned envelope on-site to confirm the patient's group allocation. The study implemented a double-blind design, wherein the patients, researchers, and outcome assessors remained blinded to group assignment until data lock.

### 2.3. Cohort Information

All the participants in this study were CHB patients under the long period of regular treatment, meeting the aim of our study for the influence of accompanied dietary self-management education in the courses. Patients in both groups had similar CHB conditions and received the same standard clinical treatments. The privacy information of the patients was hidden, and the statistical basic information is listed in Table 2. The cohort establishment was unbiased, comprehensive, voluntary, and random. Patients with comorbidities, such as diabetes and hypertension, were discreetly and randomly divided into two groups. The counts between the refined group and the control group were relatively equal, and the ratio of subgroups was rational (see Table 1).

### 2.4. Self-Management Education

The observation period started after recruitment, and the evaluation was conducted after 6 months of regular treatment and clinical care. During the first 2 months, participants received two face-to-face individual conversations for at least 30 min from a nursing researcher for the dietary self-management education. During the last 4 months, they received around 20 min of telephone educational communication from the researcher once in a month. In the first conversation, the researcher stated the risk factors for the participant according to the physical conditions, psychosocial information, dietary habits, and daily activities. Then, the researcher taught the participant a self-management program based on the traditional principles such as a higher protein and vitamin diet and prevention of fatigue, quitting smoking, alcohol avoidance, and stress management. Subsequently, the researcher guided the participant to set a healthy schedule including the management of diet, exercise, and other daily life activities, for the following months, to achieve the long-term goal of this program.

### 2.5. Study Oversight

This study was conducted with the explicit acknowledgment of all participants, and the written informed consent was obtained from each of them before recruitment. The Ethics Committee of the Nantong Third People's Hospital approved the study. All authors of this study affirm the integrity and accuracy of the data collected, as well as the strict adherence to the study protocol.

### 2.6. Assessments

The assessment of life quality was conducted using the score method following the social function (SF)-36 survey items for each patient. The score of 100 was considered as the best and 0 as the worst for each item, including physical functioning (PF), role physical (RP), role emotional (RE), mental health (MH), SF, vitality (VT), general health (GH), and bodily pain (BP). The assessment of perceived risk questionnaire (PRQ) was according to responses of the patients from the four questions, containing the aggravation risks, seriousness cognition, significance of the education, and the self-control difficulty, with 5 scores for each question.

### 2.7. Statistical Analysis

All comparison data were statistically analyzed by Student's t-test or one-way ANOVA with the software GraphPad Prism 8 to compare average values between two different groups. *p* values less than 0.05 were deemed statistically significantly different, and the error bars were presented as the SEM statistical results. The basic information of the samples was analyzed by the chi-square test, and the *p* values reflected the degrees of freedom.

## 3. Results

### 3.1. Characteristics of the Participants

Ninety participants were unbiasedly enrolled together, being divided into either the refined group or the control group (see Table 2). All of the participants were HBsAg-positive as the CHB patients. The indices of ALT, AST, and TBIL were set as the primary endpoint to meet our goal of investigating whether the vegetable oil dietary self-management was beneficial for liver recovery in CHB patients. The baseline of ALT in the refined group and the control group were 96.31 ± 4.15 U/L and 90.34 ± 4.42 U/L, respectively. Furthermore, the baseline of AST in the two groups was 112.08 ± 6.63 U/L and 123.54 ± 6.51 U/L, and the baseline of TBIL was 80.22 ± 3.38 μmol/L and 81.72 ± 3.01 μmol/L, respectively (see Table 2).

Of the participants in the refined group, 5 had the accompanying disease of diabetes and three had hypertension. Correspondingly, three individuals accompanied with diabetes and two with hypertension were included in the control group. The remaining individuals without the accompanying disease were 7 in the refined group and 40 in the control group, presenting relatively average in general (see Table 2).

### 3.2. Response of Dietary Self-Management Refinement

**Figure 2:**
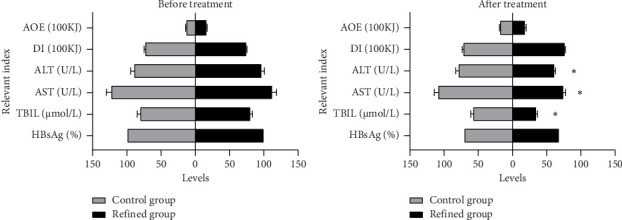
The relevant indicators of liver function or physiological function.

After 6 months of regular clinical treatment combined with the personalized dietary self-management, the patients in the refined group had index reduction in ALT, AST, and TBIL from 96.31 (U/L), 112.08 (U/L), and 80.22 (μmol/L) to 61.01 (U/L), 75.26 (U/L), and 35.37 (μmol/L) in average, respectively. Correspondingly, the ALT, AST, and TBIL indexes in patients who educated themselves with traditional dietary self-management had decreased from the average values of 90.34 (U/L), 123.54 (U/L), and 81.72 (μmol/L) to the values in 6 months after as 89.03, 109.23, and 58.08 in average, respectively (see Figure 2 and Table 3). For the amount of exercise (AOE), the average values in the refined group and control group were both increased from 1615.56 kJ vs. 1437.47 kJ to 1932.31 kJ vs. 1877.58 kJ. The average value of dietary intake (DI) was relatively stable in both groups, and it changed from 7389.98 kJ to 7700.64 kJ after 6 months in the refined group and from 7411.11 kJ to 7183.87 kJ in the control group. Moreover, the positive ratios of HBsAg in both groups declined, as the final ratio of 68.89% vs. 71.11%, cutoff at the sixth month, which did not show significant differences in the HBsAg clearance rate between the two groups (see Figure 2 and Table 3).

### 3.3. Life Qualitative Evaluation and Perceived Risk Questionnaire Scores

**Figure 3:**
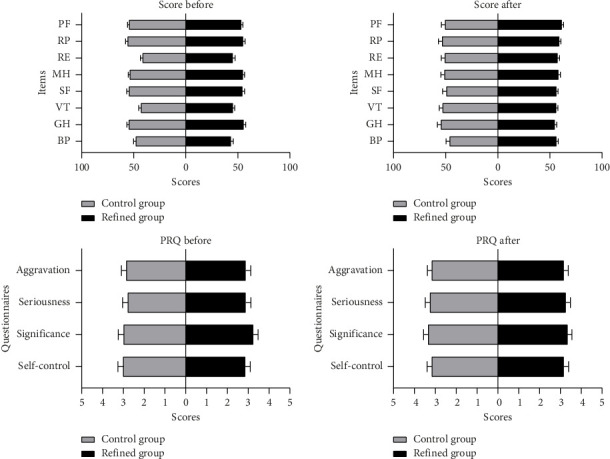
Scores of life quality and perceived risk questionnaire.

The baseline of life qualitative evaluation was similar between the refined group and control group; three items including RE, VT, and BP were relatively lower, as the scores were less than 50 (100 scores for full). After 6 months of regular clinical treatment combined with dietary self-management, the scores of RE, VT, and BP rose in both groups (see Figure 3 and Table 3). Generally, however, the overall trend of life qualitative evaluation in the refined group was slightly better than that of the control group, for the scores in the refined group were all above 50; while in the control group, they ranged around 50 (see Figure 3 and Table 3). For the perceived risk questionnaire scores, the differences between the two groups and the changes from the recruitment to the cutoff point were unapparent. While the general trends of scores were slightly increased in both groups after 6 months (see Figure 3 and Table 3).

## 4. Discussion

In recent years, multiple applications of combined or extended treatment strategies had raised the disease control rate of CHB [18–21]. Among those clinical attempts, the clearance ratio of HBsAg represented the only endpoint of a successful trial [18, 22, 23]. However, for the majority of the CHB patients who had not been totally cured, the improvement of their life quality by daily self-management of dietary diet should be equally important as the HBsAg clearance during the long period of treatment.

Therefore, we refined the schedule of CHB patients' diet to be more individualized, especially in the oil intake aspect of using the healthier vegetable oil, rather than following the rules to recommend all patients to cut down their oil intake. With this refinement, we observed that the personalized self-management with vegetable oil diet indeed benefited the cohort of CHB participants.

Compared to the control group, patients in the refined group had a greater reduction in the indexes of ALT, AST, and TBIL after 6 months of clinical care, which indicated that the newly personalized dietary self-management contributed to the recovery of the liver organs. On the other hand, the HBsAg clearance rate was not obviously different between the two groups, which indicated that whether the new dietary self-management project interferes with the drug functions on CHB treatment or affects the disease directly should have further investigation and discussion. The values of DI were relatively stable (did not show statistical differences at the point of sixth month compared to the primary baseline) in both the control group and the refined group (see Tables 1 and 3).

We were interested in this finding and then re-checked the past cases and screened out 54 cases with similar situations. Then we re-contacted the 54 past cases and divided them into three groups following the dietary habit in their daily life, preferring using vegetable oil in their cooking for Group 1 (23 cases), preferring using animal oil in their cooking for Group 2 (19 cases), and preferring fat-free meal or cooking without oil for Group 3 (12 cases). As a result, patients in Group 1 showed a better condition of liver recovery than those in the other two groups. The data demonstrated that the liver function relevant indicators, ALT, AST, and TBIL, had larger decreases in the patients of Group 1 (see Figure S1). For the 90 recruited patients, we had re-contacted 82 out of them (they remained on the dietary self-management) and performed the extra detection of ALT, AST, and TBIL at a prolonged time point (approximately 4–5 months) after the observation study. The results showed that the values of ALT, AST, and TBIL in both groups were decreased again. While the indexes of ALT, AST, and TBIL in the refined group were still lower than those in the control group (Figure S2). These data were consistent with the results in our observational study and indirectly supported the benefits of our dietary self-management education and the value of vegetable oil as a better way of energy intake during the CHB treatments.

For some basic information of the patients, correlation analysis has been performed in advance and indicated that the marital status and living situation were not the correlating factors to ALT, AST, or TBIL, though they presented statistical differences between the two groups (see Table S1). On the other hand, as the correlation analysis was performed in this small sample group, the information was relatively lower in accuracy than that in the large cohort and might be immature for multivariate regression analysis. Therefore, a larger scale of the trial and extending the studies by family doctors and community nurses are needed for further investigation in the future.

Overall, these results demonstrated that the refined personalized dietary self-management had better efficacy on accelerating the liver recovery in CHB patients, having the potential to be extended in a larger cohort for exploring its worth in clinical care.

## Figures and Tables

**Table 1:** The personalized dietary self-management plan for the participants.

CHB patient's condition	Dietary self-management	Meals per day	Note
Fatter body size	Low-oil combined high-diet-fiber diet with skimmed milk	3–5	The dietary self-management was refined based on the traditional diet for CHB patients including higher protein and normal vitamin intake
Medium body size	Normal diet with vegetable oil and skimmed milk	3–5	
Thinner body size	Normal diet with vegetable oil and whole milk	3–5	
Accompanied with diabetes	Low-oil combined with low-carbohydrate diet	4–6	
Accompanied with hypertension	Low-oil combined with low-sodium and higher potassium diet	3–5	

**Table 2:** The basic information and baseline of CHB patients.

Variable		Refined group	Control group	*p* value
Gender	Female	24	20	0.2301
	Male	21	25	

Marriage	Married	39	27	0.0002
	Unmarried	6	18	

Living situation	Live alone	26	15	0.0021
	Cohabitation with relatives	15	25	
	Cohabitation without relatives	4	5	

Place of residence	City center	27	30	0.4065
	Outskirts	9	9	
	Village	9	6	

Education	Primary school or below	9	8	0.0574
	Senior high school	17	10	
	College degree	10	13	
	Bachelor's degree or above	9	14	

Income (CNY per month)	< 3000	9	6	0.1883
	3000–4999	22	22	
	5000–9999	13	12	
	> 10,000	1	5	

Accompanied disease	Diabetes	5	3	0.3573
	Hypertension	3	2	
	None	37	40	

Physique	Fatter size	15	16	0.5761
	Medium size	16	18	
	Thinner size	14	11	

Age		52.29 ± 3.03	53.47 ± 3.12	0.7661

ALT (U/L)		96.31 ± 4.15	90.34 ± 4.42	< 0.0001

AST (U/L)		112.08 ± 6.63	123.54 ± 6.51	< 0.0001

TBIL (μmol/L)		80.22 ± 3.38	81.72 ± 3.01	< 0.0001

**Table 3:** Changes after 6 months of CHB treatment combined with clinical care and the differences between the two groups.

Variable	Refined group			Control group		
	Before	After	*p* value	Before	After	*p* value
Amount of exercise (kJ)	1615.56 ± 63.38	1932.31 ± 84.66	0.00393	1437.47 ± 54.83	1877.58 ± 30.7	< 0.00001
Dietary intake (kJ)	7389.98 ± 78.28	7700.64 ± 76.46	0.00615	7411.11 ± 72.69	7183.87 ± 111.5	0.09492
ALT (U/L)	96.31 ± 4.15	61 ± 2.33	< 0.00001	90.34 ± 4.42	89.03 ± 3.56	0.82016
AST (U/L)	112.08 ± 6.63	75.26 ± 2.43	< 0.00001	123.54 ± 6.51	109.23 ± 5.22	0.09331
TBIL (μmol/L)	80.22 ± 3.38	35.37 ± 1.32	< 0.00001	81.72 ± 3.01	58.08 ± 2.77	< 0.00001
HBsAg clearance rate (%)	0	31.11 (14/45)	—	0	28.89 (13/45)	—
Score of dietary self-management	18.09 ± 0.86	19.38 ± 0.8	0.2809	18.24 ± 0.92	18.47 ± 0.83	0.02948
Physical functioning score (PF)	53.93 ± 0.92	62.07 ± 1.09	< 0.00001	55.09 ± 0.82	48.18 ± 1.43	0.00008
Role physical score (RP)	55.67 ± 1.28	59.6 ± 0.85	0.01296	56.47 ± 1.34	42.04 ± 1.89	< 0.00001
Role emotional score (RE)	45.84 ± 1.46	58.11 ± 1.21	< 0.00001	42.22 ± 1.31	47.31 ± 1.47	0.01228
Mental health score (MH)	55.58 ± 0.91	58.87 ± 1.14	0.028	54.42 ± 0.85	49.82 ± 1.4	0.00669
Social function score (SF)	55.02 ± 1.36	56.91 ± 0.95	0.26436	55.27 ± 1.37	47.04 ± 1.7	0.00035
Vitality score (VT)	45.96 ± 1.15	56.67 ± 1.08	< 0.00001	43.73 ± 1.39	48.64 ± 1.7	0.02965
General health score (GH)	56.2 ± 1.41	55.33 ± 1.3	0.65692	55.38 ± 1.14	49.07 ± 1.53	0.00154
Bodily pain score (BP)	44.09 ± 1.49	56.93 ± 1.12	< 0.00001	48.73 ± 1.34	46.89 ± 1.44	0.3563
PRQ-aggravation	2.91 ± 0.21	3.2 ± 0.18	0.30442	2.98 ± 0.22	2.89 ± 0.2	0.76721
PRQ-seriousness	2.91 ± 0.22	3.29 ± 0.19	0.19922	3.04 ± 0.2	2.82 ± 0.21	0.44277
PRQ-significance	3.27 ± 0.2	3.38 ± 0.18	0.68681	2.91 ± 0.22	3.02 ± 0.22	0.72389
PRQ-self-control	2.89 ± 0.2	3.2 ± 0.19	0.2671	2.53 ± 0.18	3.04 ± 0.22	0.0802

## Data Availability

The data that support the findings of this study are available from the corresponding author upon reasonable request.

## Supporting Information

Figure S1: Changes of ALT, AST, and TBIL in patients of Groups 1, 2, and 3 before and after CHB treatments.

Figure S2: Comparison of ALT, AST, and TBIL detected results in the re-contacted patients from the refined group and control group.

Table S1: Correlation analysis of multiple factors versus ALT, AST, and TBIL.
