# Supplementary material for: Personalized Dietary Self-Management and Its Influence on Disease Progression in Chronic Hepatitis B
Source: J Nutr Metab. 2025 Jul 17;2025:5585004. doi: 10.1155/jnme/5585004 (PMC12289363; doi:10.1155/jnme/5585004)
Supplement: Supporting Information — Additional supporting information can be found online in the Supporting Information section. [file 5585004.f1.zip › Supplementary materials.docx]

**Supplementary Figure Legend**

**Figure S1.**

Changes of ALT, AST and TBIL in patients of group 1, 2 and 3 before and after CHB treatments.

**Figure S2.**

Comparison of ALT, AST and TBIL detected results in the re-contacted patients from the refined group and control group.

**Supplementary Table Legend**

**Table S1.**

Correlation analysis of multiple factors versus ALT, AST and TBIL.

**Figure S1**

**
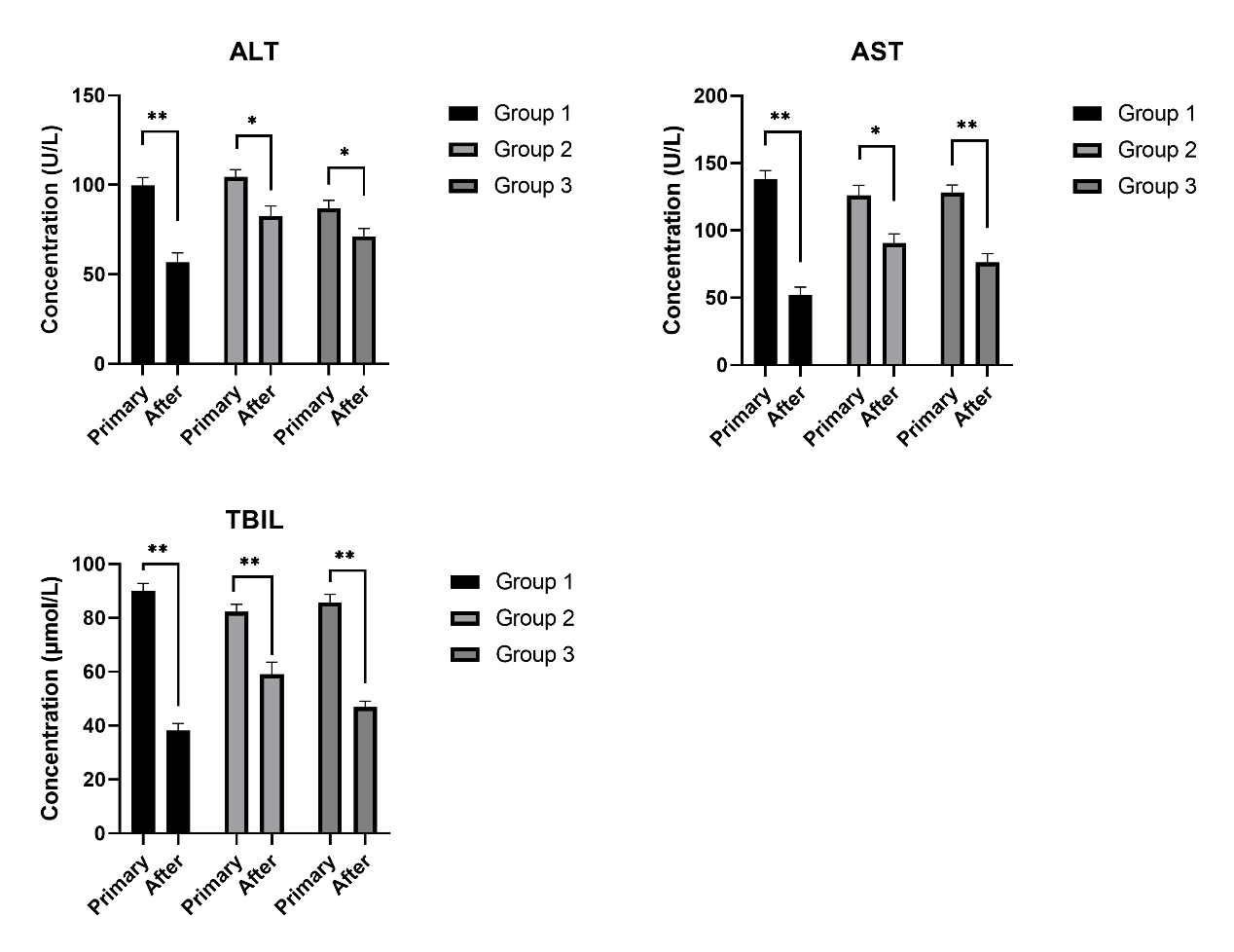
**

**Figure S2**

**
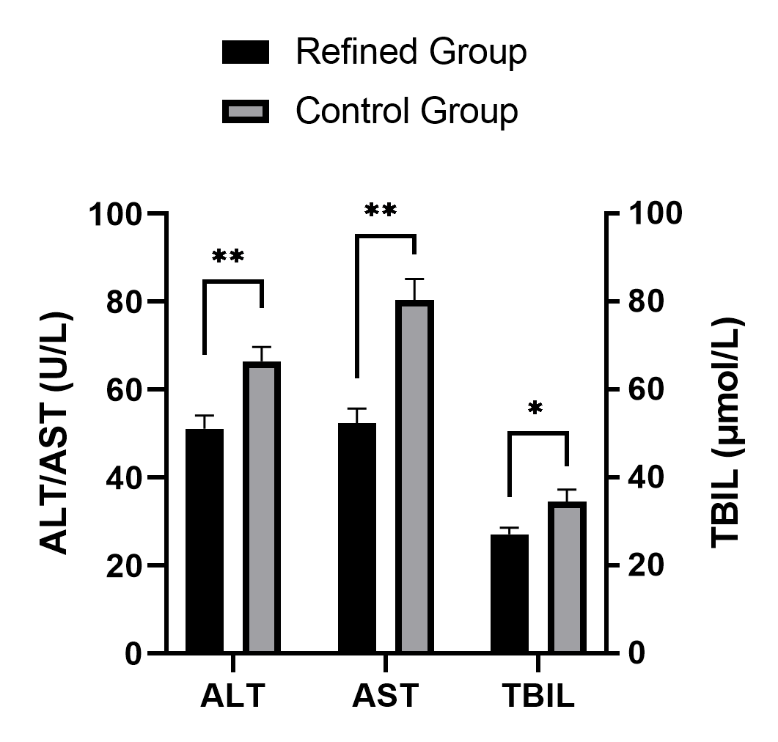
**

**Table S1**

| **ALT** | **Spearman r** | **r** | **95% confidence interval** | **P (two-tailed)** | **P value summary** | **Exact or approximate P value?** | **Significant? (alpha = 0.05)** | **Number of XY Pairs** |
| --- | --- | --- | --- | --- | --- | --- | --- | --- |
| ALT (U/L) vs.gender |  | -0.1818 | -0.4583 to 0.1268 | 0.232 | ns | Approximate | No | 45 |
| ALT (U/L) vs.age |  | 0.1242 | -0.1844 to 0.4105 | 0.4163 | ns | Approximate | No | 45 |
| ALT (U/L) vs.marriage |  | -0.06041 | -0.3556 to 0.2458 | 0.6934 | ns | Approximate | No | 45 |
| ALT (U/L) vs.Living situation |  | 0.06499 | -0.2414 to 0.3596 | 0.6715 | ns | Approximate | No | 45 |
| ALT (U/L) vs.Place residence |  | -0.2239 | -0.4923 to 0.08343 | 0.1393 | ns | Approximate | No | 45 |
| ALT (U/L) vs.Education |  | -0.2546 | -0.5166 to 0.05096 | 0.0914 | ns | Approximate | No | 45 |
| ALT (U/L) vs.Income (CNY per month) |  | -0.1763 | -0.4538 to 0.1324 | 0.2467 | ns | Approximate | No | 45 |
| ALT (U/L) vs.Accompanied disease |  | 0.2407 | -0.06575 to 0.5057 | 0.1112 | ns | Approximate | No | 45 |
| ALT (U/L) vs. Amount of exercise |  | 0.005435 | -0.2967 to 0.3066 | 0.9717 | ns | Approximate | No | 45 |
| ALT (U/L) vs. Dietary intake (kJ) |  | -0.01245 | -0.3130 to 0.2903 | 0.9353 | ns | Approximate | No | 45 |
| ALT (U/L) vs. smoke |  | 0.1194 | -0.1891 to 0.4065 | 0.4346 | ns | Approximate | No | 45 |
| ALT (U/L) vs. drink |  | 0.0234 | -0.2803 to 0.3228 | 0.8787 | ns | Approximate | No | 45 |
| ALT (U/L) vs. Staying up late |  | -0.01221 | -0.3127 to 0.2905 | 0.9366 | ns | Approximate | No | 45 |
| ALT (U/L) |  |  |  |  |  |  |  |  |
| ALT (U/L) vs. AST (U/L) |  | 0.03235 | -0.2720 to 0.3308 | 0.8329 | ns | Approximate | No | 45 |
| ALT (U/L) vs. TBIL (μmol/L) |  | -0.08492 | -0.3769 to 0.2225 | 0.5791 | ns | Approximate | No | 45 |
| ALT (U/L) vs. HBsAg clearance rate (%) | Horizontal line |  |  |  |  |  |  | 45 |
| ALT (U/L) vs. Score of dietary self-management |  | -0.4412 | -0.6556 to -0.1609 | 0.0024 | ** | Approximate | Yes | 45 |
| ALT (U/L) vs. Physical functioning Score (PF) |  | 0.07974 | -0.2274 to 0.3725 | 0.6026 | ns | Approximate | No | 45 |
| ALT (U/L) vs. Role physical Score (RP) |  | 0.1634 | -0.1454 to 0.4433 | 0.2834 | ns | Approximate | No | 45 |
| ALT (U/L) vs. Role emotional Score (RE) |  | -0.1928 | -0.4673 to 0.1156 | 0.2044 | ns | Approximate | No | 45 |
| ALT (U/L) vs. Mental health Score (MH) |  | 0.1555 | -0.1534 to 0.4367 | 0.3078 | ns | Approximate | No | 45 |
| ALT (U/L) vs. Social function Score (SF) |  | 0.03836 | -0.2664 to 0.3361 | 0.8025 | ns | Approximate | No | 45 |
| ALT (U/L) vs. Vitality Score (VT) |  | -0.1531 | -0.4347 to 0.1558 | 0.3154 | ns | Approximate | No | 45 |
| ALT (U/L) vs. General health Score (GH) |  | 0.2353 | -0.07146 to 0.5014 | 0.1197 | ns | Approximate | No | 45 |
| ALT (U/L) vs. Bodily pain Score (BP) |  | -0.162 | -0.4420 to 0.1469 | 0.2878 | ns | Approximate | No | 45 |
| ALT (U/L) vs. PRQ - Aggravation |  | -0.1839 | -0.4601 to 0.1247 | 0.2265 | ns | Approximate | No | 45 |
| ALT (U/L) vs. PRQ - Seriousness |  | -0.172 | -0.4503 to 0.1368 | 0.2586 | ns | Approximate | No | 45 |
| ALT (U/L) vs. PRQ - Significance |  | -0.01309 | -0.3135 to 0.2897 | 0.932 | ns | Approximate | No | 45 |
| ALT (U/L) vs. PRQ - Self-control |  | -0.1088 | -0.3974 to 0.1995 | 0.477 | ns | Approximate | No | 45 |

| **AST** | **Spearman r** | **r** | **95% confidence interval** | **P (two-tailed)** | **P value summary** | **Exact or approximate P value?** | **Significant? (alpha = 0.05)** | **Number of XY Pairs** |
| --- | --- | --- | --- | --- | --- | --- | --- | --- |
| AST (U/L) vs.gender |  | -0.05831 | -0.3538 to 0.2477 | 0.7036 | ns | Approximate | No | 45 |
| AST (U/L) vs.age |  | 0.01423 | -0.2887 to 0.3146 | 0.9261 | ns | Approximate | No | 45 |
| AST (U/L) vs.marriage |  | 0.09564 | -0.2122 to 0.3862 | 0.532 | ns | Approximate | No | 45 |
| AST (U/L) vs.Living situation |  | 0.2291 | -0.07797 to 0.4965 | 0.1301 | ns | Approximate | No | 45 |
| AST (U/L) vs.Place residence |  | -0.05005 | -0.3465 to 0.2555 | 0.744 | ns | Approximate | No | 45 |
| AST (U/L) vs.Education |  | 0.02542 | -0.2784 to 0.3246 | 0.8684 | ns | Approximate | No | 45 |
| AST (U/L) vs.Income (CNY per month) |  | 0.01157 | -0.2911 to 0.3122 | 0.9399 | ns | Approximate | No | 45 |
| AST (U/L) vs.Accompanied disease |  | -0.1105 | -0.3989 to 0.1978 | 0.4699 | ns | Approximate | No | 45 |
| AST (U/L) vs. Amount of exercise |  | -0.3949 | -0.6224 to -0.1058 | 0.0073 | ** | Approximate | Yes | 45 |
| AST (U/L) vs. Dietary intake (kJ) |  | 0.05317 | -0.2526 to 0.3492 | 0.7287 | ns | Approximate | No | 45 |
| AST (U/L) vs. smoke |  | 0.002628 | -0.2993 to 0.3041 | 0.9863 | ns | Approximate | No | 45 |
| AST (U/L) vs. drink |  | -0.03664 | -0.3346 to 0.2680 | 0.8111 | ns | Approximate | No | 45 |
| AST (U/L) vs. Staying up late |  | 0.217 | -0.09067 to 0.4868 | 0.1523 | ns | Approximate | No | 45 |
| AST (U/L) vs. ALT (U/L) |  | 0.03235 | -0.2720 to 0.3308 | 0.8329 | ns | Approximate | No | 45 |
| AST (U/L) |  |  |  |  |  |  |  | 45 |
| AST (U/L) vs. TBIL (μmol/L) |  | 0.1114 | -0.1969 to 0.3997 | 0.4663 | ns | Approximate | No | 45 |
| AST (U/L) vs. HBsAg clearance rate (%) | Horizontal line |  |  |  |  |  |  | 45 |
| AST (U/L) vs. Score of dietary self-management |  | 0.063 | -0.2433 to 0.3579 | 0.681 | ns | Approximate | No | 45 |
| AST (U/L) vs. Physical functioning Score (PF) |  | -0.09036 | -0.3816 to 0.2172 | 0.555 | ns | Approximate | No | 45 |
| AST (U/L) vs. Role physical Score (RP) |  | 0.2216 | -0.08581 to 0.4905 | 0.1435 | ns | Approximate | No | 45 |
| AST (U/L) vs. Role emotional Score (RE) |  | -0.1572 | -0.4381 to 0.1516 | 0.3023 | ns | Approximate | No | 45 |
| AST (U/L) vs. Mental health Score (MH) |  | 0.1872 | -0.1214 to 0.4627 | 0.2183 | ns | Approximate | No | 45 |
| AST (U/L) vs. Social function Score (SF) |  | 0.3242 | 0.02492 to 0.5701 | 0.0298 | * | Approximate | Yes | 45 |
| AST (U/L) vs. Vitality Score (VT) |  | -0.08946 | -0.3809 to 0.2181 | 0.5589 | ns | Approximate | No | 45 |
| AST (U/L) vs. General health Score (GH) |  | 0.032 | -0.2723 to 0.3305 | 0.8347 | ns | Approximate | No | 45 |
| AST (U/L) vs. Bodily pain Score (BP) |  | 0.2129 | -0.09492 to 0.4835 | 0.1604 | ns | Approximate | No | 45 |
| AST (U/L) vs. PRQ - Aggravation |  | 0.01823 | -0.2850 to 0.3182 | 0.9054 | ns | Approximate | No | 45 |
| AST (U/L) vs. PRQ - Seriousness |  | -0.1513 | -0.4332 to 0.1576 | 0.3212 | ns | Approximate | No | 45 |
| AST (U/L) vs. PRQ - Significance |  | 0.06468 | -0.2417 to 0.3593 | 0.6729 | ns | Approximate | No | 45 |
| AST (U/L) vs. PRQ - Self-control |  | -0.2195 | -0.4888 to 0.08805 | 0.1475 | ns | Approximate | No | 45 |

| **TBIL** | **Spearman r** | **r** | **95% confidence interval** | **P (two-tailed)** | **P value summary** | **Exact or approximate P value?** | **Significant? (alpha = 0.05)** | **Number of XY Pairs** |
| --- | --- | --- | --- | --- | --- | --- | --- | --- |
| TBIL (μmol/L) vs.gender |  | -0.005145 | -0.3064 to 0.2970 | 0.9732 | ns | Approximate | No | 45 |
| TBIL (μmol/L) vs.age |  | 0.0759 | -0.2311 to 0.3691 | 0.6202 | ns | Approximate | No | 45 |
| TBIL (μmol/L) vs.marriage |  | -0.07551 | -0.3688 to 0.2314 | 0.622 | ns | Approximate | No | 45 |
| TBIL (μmol/L) vs.Living situation |  | 0.1775 | -0.1312 to 0.4548 | 0.2434 | ns | Approximate | No | 45 |
| TBIL (μmol/L) vs.Place residence |  | -0.07576 | -0.3690 to 0.2312 | 0.6209 | ns | Approximate | No | 45 |
| TBIL (μmol/L) vs.Education |  | 0.01621 | -0.2869 to 0.3163 | 0.9158 | ns | Approximate | No | 45 |
| TBIL (μmol/L) vs.Income (CNY per month) |  | 0.04776 | -0.2576 to 0.3445 | 0.7554 | ns | Approximate | No | 45 |
| TBIL (μmol/L) vs.Accompanied disease |  | 0.2782 | -0.02560 to 0.5350 | 0.0642 | ns | Approximate | No | 45 |
| TBIL (μmol/L) vs. Amount of exercise |  | -0.1527 | -0.4343 to 0.1562 | 0.3168 | ns | Approximate | No | 45 |
| TBIL (μmol/L) vs. Dietary intake (kJ) |  | 0.06997 | -0.2367 to 0.3640 | 0.6479 | ns | Approximate | No | 45 |
| TBIL (μmol/L) vs. smoke |  | 0.1168 | -0.1917 to 0.4042 | 0.445 | ns | Approximate | No | 45 |
| TBIL (μmol/L) vs. drink |  | -0.1764 | -0.4539 to 0.1323 | 0.2464 | ns | Approximate | No | 45 |
| TBIL (μmol/L) vs. Staying up late |  | 0.08247 | -0.2248 to 0.3748 | 0.5902 | ns | Approximate | No | 45 |
| TBIL (μmol/L) vs. ALT (U/L) |  | -0.08492 | -0.3769 to 0.2225 | 0.5791 | ns | Approximate | No | 45 |
| TBIL (μmol/L) vs. AST (U/L) |  | 0.1114 | -0.1969 to 0.3997 | 0.4663 | ns | Approximate | No | 45 |
| TBIL (μmol/L) |  |  |  |  |  |  |  | 45 |
| TBIL (μmol/L) vs. HBsAg clearance rate (%) | Horizontal line |  |  |  |  |  |  | 45 |
| TBIL (μmol/L) vs. Score of dietary self-management |  | -0.07591 | -0.3691 to 0.2311 | 0.6202 | ns | Approximate | No | 45 |
| TBIL (μmol/L) vs. Physical functioning Score (PF) |  | -0.08479 | -0.3768 to 0.2226 | 0.5797 | ns | Approximate | No | 45 |
| TBIL (μmol/L) vs. Role physical Score (RP) |  | 0.03141 | -0.2729 to 0.3300 | 0.8377 | ns | Approximate | No | 45 |
| TBIL (μmol/L) vs. Role emotional Score (RE) |  | -0.0648 | -0.3595 to 0.2416 | 0.6724 | ns | Approximate | No | 45 |
| TBIL (μmol/L) vs. Mental health Score (MH) |  | -0.01146 | -0.3121 to 0.2912 | 0.9404 | ns | Approximate | No | 45 |
| TBIL (μmol/L) vs. Social function Score (SF) |  | -0.0524 | -0.3486 to 0.2533 | 0.7324 | ns | Approximate | No | 45 |
| TBIL (μmol/L) vs. Vitality Score (VT) |  | 0.1981 | -0.1102 to 0.4716 | 0.1922 | ns | Approximate | No | 45 |
| TBIL (μmol/L) vs. General health Score (GH) |  | 0.1413 | -0.1675 to 0.4249 | 0.3544 | ns | Approximate | No | 45 |
| TBIL (μmol/L) vs. Bodily pain Score (BP) |  | 0.2503 | -0.05563 to 0.5132 | 0.0973 | ns | Approximate | No | 45 |
| TBIL (μmol/L) vs. PRQ - Aggravation |  | 0.1671 | -0.1417 to 0.4463 | 0.2726 | ns | Approximate | No | 45 |
| TBIL (μmol/L) vs. PRQ - Seriousness |  | 0.02667 | -0.2772 to 0.3257 | 0.8619 | ns | Approximate | No | 45 |
| TBIL (μmol/L) vs. PRQ - Significance |  | 0.183 | -0.1256 to 0.4593 | 0.2289 | ns | Approximate | No | 45 |
| TBIL (μmol/L) vs. PRQ - Self-control |  | -0.02792 | -0.3269 to 0.2761 | 0.8555 | ns | Approximate | No | 45 |
